# A Small Molecule Swertisin from *Enicostemma littorale* Differentiates NIH3T3 Cells into Islet-Like Clusters and Restores Normoglycemia upon Transplantation in Diabetic Balb/c Mice

**DOI:** 10.1155/2013/280392

**Published:** 2013-04-15

**Authors:** Nidheesh Dadheech, Sanket Soni, Abhay Srivastava, Sucheta Dadheech, Shivika Gupta, Renjitha Gopurappilly, Ramesh R. Bhonde, Sarita Gupta

**Affiliations:** ^1^Department of Biochemistry, Faculty of Science, The M.S. University of Baroda, Vadodara Gujarat, 390 002, India; ^2^Hislope College of Biotechnology, Nagpur University, Nagpur, Maharashtra, India; ^3^Manipal Institute of Regenerative Medicine, Manipal University, Bangalore, Karnataka, 560 065, India

## Abstract

*Aim*. Stem cell therapy is one of the upcoming therapies for the treatment of diabetes. Discovery of potent differentiating agents is a prerequisite for increasing islet mass. The present study is an attempt to screen the potential of novel small biomolecules for their differentiating property into pancreatic islet cells using NIH3T3, as representative of extra pancreatic stem cells/progenitors. *Methods*. To identify new agents that stimulate islet differentiation, we screened various compounds isolated from *Enicostemma littorale* using NIH3T3 cells and morphological changes were observed. Characterization was performed by semiquantitative RT-PCR, Q-PCR, immunocytochemistry, immunoblotting, and insulin secretion assay for functional response in newly generated islet-like cell clusters (ILCC). Reversal of hyperglycemia was monitored after transplanting ILCC in STZ-induced diabetic mice. *Results*. Among various compounds tested, swertisin, an isolated flavonoid, was the most effective in differentiating NIH3T3 into endocrine cells. Swertisin efficiently changed the morphology of NIH3T3 cells from fibroblastic to round aggregate cell cluster in huge numbers. Dithizone (DTZ) stain primarily confirmed differentiation and gene expression studies signified rapid onset of differentiation signaling cascade in swertisin-induced ILCC. Molecular imaging and immunoblotting further confirmed presence of islet specific proteins. Moreover, glucose induced insulin release (*in vitro*) and decreased fasting blood glucose (FBG) (*in vivo*) in transplanted diabetic BALB/c mice depicted functional maturity of ILCC. Insulin and glucagon expression in excised islet grafts illustrated survival and functional integrity. *Conclusions*. Rapid induction for islet differentiation by swertisin, a novel herbal biomolecule, provides low cost and readily available differentiating agent that can be translated as a therapeutic tool for effective treatment in diabetes.

## 1. Introduction

Diabetes is a devastating disease, affecting millions of people worldwide. Hyperglycemia is a principal signature of both type 1 diabetes (T1D) and type 2 diabetes (T2D). Reversal of hyperglycemia by exogenous insulin may delay or attenuate but never eliminate the risk for developing secondary complications [1]. Islet transplantation is a modern approach that has become more prevalent in clinics nowadays. It offers internal glucose homeostasis with low surgery risk and reduces complications in diabetic patients. However, islets derived from multiple donors require immunosuppressors. Also inadequate islet supply from cadaveric pancreas has limited the widespread utilization of this approach [2]. 

Cell-based therapy, principally new islets derived from stem cell differentiation, is a new area of research in diabetes. Recent studies have shown that embryonic stem cells, induced pluripotent stem cells, adult bone marrow mesenchymal stem cells, and many other tissue-specific progenitors have the ability to convert into cell of multiple lineages like blood, liver, lung, skin, cardiac, muscles, and neurons including insulin producing *β* cells upon appropriate induction [3, 4]. Although few reports have shown generation of islet mass from various pluripotent and multipotent stem cells, progress has been hampered in increasing differentiated islet yield due to lack of potent and economical islet differentiating agents. Success in exploring various adult tissues to isolate stem cell population has been recorded for autologous transplants. However, not much work has been done for identification of potent differentiating agents that not only accelerate the rate of differentiation but also produce functional cell types in large numbers. If this can be achieved, the utilization of huge islet mass seems to be feasible to withstand the shortage of autologous islet transplant in near future and prevent diabetes and its complications. 

There are many growth factors and differentiating agents known to promote differentiation or regeneration of pancreatic *β* cells [5]. These include nicotinamide, glucagon-like peptide, gastrin, activin A, betacellulin, Reg protein, INGAP, and hepatocyte growth factor (HGF). Practically none of them were translated as therapeutic molecule for islet generation and transplant in clinics, as all of them are associated with high cost and low yield of islet clusters. 

Plants are exemplary sources of medicinal values, and an important thing is to properly identify and screen for their miraculous properties. From a practical point of view, low molecular weight compounds are favorable for such cellular interaction studies, because such agents are not immunogenic and may be effective even when administered orally. Numerous plant products have demonstrated antidiabetic activity [6–9]. Reports have appeared in recent times regarding inductive agents that have been shown to stimulate regeneration and replenishment of islet cells from herbal sources. Kojima and Umezawa demonstrated islet differentiation activity of conophylline molecule isolated from *Ervatamia microphylla* with AR42J cells, where they reported conophylline to have activin-A like activity and showed acinar to islet cell transdifferentiation [10]. On similar lines, our group has also reported antidiabetic activity of *Enicostemma littorale* Blume. 

**Figure 1:**
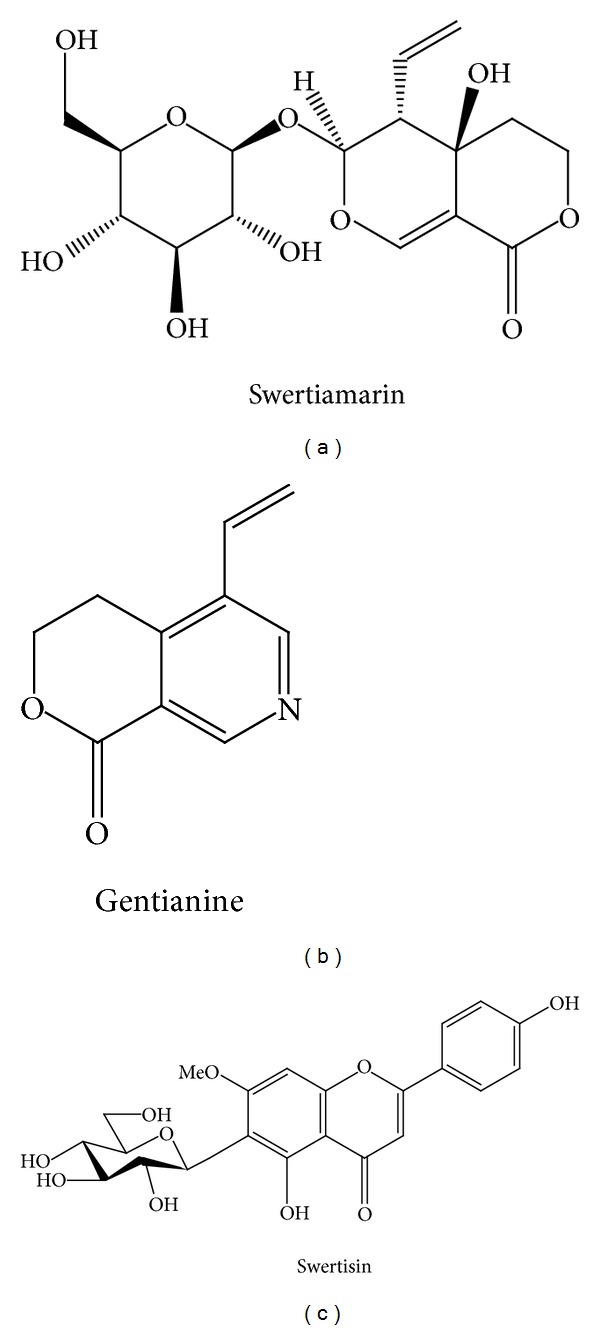
Structure of various *E. littorale* compounds: (a) represents structure of swertiamarin, (b) represents structure of Gentianine, and (c) represents structure of Swertisin molecule.

*E. littorale* is a perennial herb, belonging to Gentianaceae family and distributed throughout India. Major chemical constitutes of the plant are swertiamarin and gentianine (Figure 1) [11, 12]. Apigenin, genkwanin, isovitexin, swertisin, saponarin, and gentiocrucine [13] are also reported to be present in minor amounts. Aqueous extract of *E. littorale* demonstrated hypoglycemic potential in alloxan-induced diabetic rats [8, 9, 14]. Hypoglycemic antioxidant with hypolipidemic potential was also reported in newly diagnosed NIDDM patients [7]. The antidiabetic effect of this plant has been reported by other workers too [15, 16]. Apart from these properties, various fractions of *E. littorale* also demonstrated cytoprotective effect in isolated islets and pancreatic regeneration in both T1D and T2D animal models. Based on these observations a preliminary study was conducted by the author's group for islet differentiation property with an active herbal compound isolated from methanolic extract of *E. littorale* [17]. Further more to identify the potent islet differentiating agent, we screened various biomolecules isolated from *E. littorale* and monitored for effective stem cell differentiation with NIH3T3 cells as representative of extra pancreatic stem/progenitor cells. A flavonoid extracted from ethyl acetate fraction was found to be the most potent in increasing islet mass out of various molecules tested. This molecule was characterized by UV, TLC, HPTLC, and mass spectra analysis which was found to be identical to swertisin, reported earlier as one of the constituents of *E. littorale* [13]. Swertisin was further assessed *in vitro* at molecular, immunological, and functional levels for confirming proper differentiation into ILCC. Glucose lowering-effect-of transplanted ILCC was monitored in streptozotocin-(STZ) induced BALB/c mice suggesting functional maturity and integrity of newly generated ILCC. 

## 2. Methods

### 2.1. Plant Material

Whole dried plant was procured from Bhavnagar district in Gujarat state, India, in the month of August after authentication from taxonomist with voucher specimen number Oza 51, 51 (a) present in the Herbarium, Department of Botany, The M.S University of Baroda, Vadodara, Gujarat, India. 

### 2.2. Isolation and Characterization of Compounds from *E. littorale*

Methanol extract of *E. littorale* was prepared as discussed in earlier reports [8]. In brief, the extract (50 mL) was dissolved in distilled water (100 mL) and transferred to a separating funnel. Successive fractionation was carried out with solvents like butanol, chloroform, and ethyl acetate (250 mL each) which lay in descending manner of polarity index. Ethyl acetate fraction yielded a pale white precipitates which were further purified by washing them with acetone whereas other compounds obtained from different fractions were confirmed as swertiamarin and gentianine. This pale yellow compound was further confirmed in terms of purity as described in earlier reported methods using UV spectrum, thin layer chromatography (TLC), high performance thin layer chromatography (HPTLC) [18], and mass fragmentation pattern using ESI-MS/MS under positive and negative ionization mode.

### 2.3. Characterization by TLC, HPTLC, Ultraviolet Spectrometry, and Mass Fragmentation Pattern

The pale yellow compound was screened with TLC, using 1 mg and dissolved in 25 mL of methanol and applied on a precoated plate with Silica gel GF254 (E. Merck). The test solution was loaded and the solvent system of ethyl acetate : methanol (8 : 2) was used for development. Plate was evaporated and visualized at UV 254 nm. For HPTLC spectrum, twenty-five mg of compound was dissolved in small aliquot of methanol and developed in the solvent system mentioned above, scanned densitometrically at 243 nm and recorded using CAMAG TLC SCANNER 3 system. One milligram of compound (swertisin) was dissolved in 1 mL of methanol and scanned for the wavelength ranging between 200 and 800 nm and absorption maxima were compared with previous reports. For mass spectral identification by ESI-MS/MS, 1 mg of this compound was dissolved in methanol and was introduced for direct fragmentation in ionization chamber in positive and negative ionization mode. The base peak was recorded and compared with the molecular weight as reported earlier [18].

### 2.4. Cell Culture Maintenance and Preparation of Differentiation Medium

NIH3T3 (generous gift from Zydus Research Centre, Ahmedabad, Gujarat, India) was maintained and cultured in high glucose DMEM complete medium with PenStrep. The differentiation medium composed of DMEM Ham's F-12 (1 : 1, 8 mM glucose) without serum, cocktail supplements of insulin 5 mg/L, transferrin 5 mg/L, selenite 5 ug/L (Sigma-Aldrich, USA) and BSA 1.5 g/L with antibiotics penicillin 25 ug/mL and streptomycin 25 ug/mL.

### 2.5. FACS Analysis

Cells were trypsinized and centrifuged, and one million cells were resuspended in 100 *μ*L of wash buffer (PBS containing 10% serum), washed twice with phosphate buffered saline (PBS) containing 1% bovine serum albumin, and then incubated with primary antibody (for details and dilution see Table 2) at 4°C for 1 h. Cells were again labeled with 100 *μ*L of secondary antibody for counter staining (for details and dilution see Table 2) and incubated for an additional 40 min at 4°C [19]. Data was recorded in three observations.

### 2.6. Comparative Evaluation of *E. littorale* Extracts and Compounds for Generation of ILCC from NIH3T3 Cells

ILCC were generated from NIH3T3 cells in a four-step protocol as described in our previous report (Figure 5) [17]. Briefly, 0.1 million cells per ml were allowed to grow till 90% confluency and prior to differentiation, trypsinized with 0.05% trypsin for 60–120 sec to slightly loosen up for migration and efficient cluster formation. Finally, cells were seeded with differentiating medium (described above) and supplemented with various extracts/purified compounds with dose of 15 *μ*g/mL (*in vitro)*, while control cells received differentiation medium alone. Medium was replenished every alternate day till 8 days. On the 8th day, ILCC were collected and observed for morphological changes. Differentiated ILCC were washed three times with ice cold PBS and stained with 10 uL of DTZ stain. Images were recorded with inverted phase contrast microscope, photographed, and evaluated for total islet yield number and size and other morphological features.

### 2.7. Immunocytochemistry and Confocal Microscopy

Comparative analysis demonstrated swertisin to possess the highest islet neogenic potential depicted by the highest ILCC yield. Hence, swertisin-induced ILCC were further investigated for differentiation markers and islet hormones by immunocytochemistry. For analysis, ILCC were collected and spun at 1200 rpm (Eppendorf 5415R) and kept for adherence on glass coverslip with high glucose DMEM complete medium for 3-4 hours. Upon attachment, clusters were immediately fixed with 3.7% paraformaldehyde solution for 20 min and then washed with 0.1 M PBS. Blocking solution was then added for 30 min to prevent nonspecific binding. Thereafter ILCC were stained with primary antibodies for insulin, C-peptide, glucagon, and PDX-1 at 4°C for 18 h, washed with 0.1 M PBS, and further incubated with secondary antibodies (for details, see Table 1) at 25°C for 1 h. ILCC were photographed with confocal microscope LSM710 (Zeiss, Germany).

### 2.8. RNA Extraction, Semiquantitative Reverse Transcriptase PCR (RT-PCR), and Quantitative Real-Time PCR (Q-PCR)

Total RNA was isolated from differentiated ILCC using TRIzol Reagent (Sigma-Aldrich, USA). Purity of RNA was confirmed by *A*_260/280_ ratio and checked for integrity. Five *µ*g of total RNA was reverse transcribed into first strand cDNA and subjected to PCR amplification for various genes as mentioned in Table 2. Gradient PCR was performed with a range of annealing temperature from 51 to 60°C. cDNA was amplified using Fermentas 2x master mix (1.5 unit Taq Polymerase, 2 mM dNTP, 10x Tris, glycerol reaction buffer, 25 mM MgCl_2_) with 20 pM forward and reverse primers (see Table 2). Gapdh served as internal control and negative RT was performed with untranscribed RNA. PCR products were separated on a 10% polyacrylamide gels (Sigma-Aldrich, USA) and visualized and images were captured with Alpha Imager software (UVP Image Analysis Software Systems, USA) for densitometric analysis.

For real-time quantitative PCR of islet specific mRNA transcripts, again 1–1.5 *μ*g reverse-transcribed cDNA template was used from each group harvested in a time-dependent differentiation manner (2–8 days) using Fermentas first strand cDNA Reverse-Transcription kit. SYBR Green reactions using SYBR Green PCR Master Mix (Fermentas Inc., USA) were assembled along with 250 nM primers according to the manufacturer's instructions and performed with an ABI 7500 real-time PCR machine using standard comparative Ct value detection programme (Applied Biosystems). Relative expression of islet genes encoded mRNA was then determined after normalization to beta actin as endogenous control. Relative quantification value (RQ value) and ΔΔCt values were then calculated using 7500 software v2.0.6 (Applied Biosystems 7500 machine, ABI). All primer sequences used were intron flanking primers, negating the possibility of false amplification from genomic DNA contamination. The details of primer sequences are shown in Table 2.

### 2.9. Immunoblotting for Islet Differentiation Proteins

ILCC were collected by centrifugation and suspended in lysis buffer (1 mM EDTA, 50 mM Tris-HCl pH 7.5, 70 mM NaCl, 1% Triton, 50 mM NaF) containing 1x proteinase inhibitors (Roche), incubated on ice for 30 min. After centrifugation at 16000 g for 15 min at 4°C, the supernatant was collected and kept at −80°C for future use. Total protein content was quantified using Bradford assay (Biolrad Bradford Solution, USA). Ten *μ*g of protein was loaded on a 12% polyacrylamide gel and then electrophoretically transferred onto a Hybond-Nitrocellulose membrane (GE Healthcare). The membrane was then incubated for 1 h at room temperature in blocking buffer (TBS-T containing 8% skimmed milk) and further incubated overnight with the primary antibody at 4°C (details in Table 1). Membrane was then washed four times with TBS-T and incubated with HPR-conjugated secondary antibody for 1 h (details in Table 1). Finally, membrane was developed and visualized with Enhanced Chemiluminscence western blotting detection system (Millipore Inc. USA).

### 2.10. Glucose Induced Insulin/C-Peptide Release Assay

Differentiated ILCC were initially incubated for 30 min in glucose-free Krebs-Ringer bicarbonate buffer (KRB) containing 0.5% bovine serum albumin and then induced with 5.5, 20 mM glucose, and 10 mM L-arginine for additional 3 hours on constant shaking condition at 5% CO_2_ and 95% O_2_. After brief centrifugation, the supernatant was collected and frozen at –70°C until further analysis. Insulin/C-peptide assay was performed using mouse-insulin ELISA (Mercodia Inc., USA) and mouse C-peptide ELISA (ALPCO Immunoassays, USA). 

### 2.11. Animal Selection and Induction of Diabetes

Male BALB/c mice of 3-4 weeks old weighing 15–20 grams were used for transplantation experiments. All animal experiments were performed in accordance with our institutional Ethical Committee for Animal Experiment and CPCSEA guidelines and regulations. Animals were kept in animal house with 12 hours light and 12 hours dark cycle and allowed to have water and pellet diet* ad libitum*. Diabetes was induced with streptozotocin injection (STZ; 65 mg/kg body weight) intraperitoneally for 5 days with overnight fasting. Diabetic status of animals was confirmed by monitoring FBG using Accu check glucometer (Accu check, Roche, USA) till 30 days for stabilization of hyperglycemia.

### 2.12. Transplantation of ILCC and Reversal of Hyperglycemia

Transplantation was performed under anesthesia with ketamine and xylazine (as per CPCSEA guidelines). Briefly, the abdomen was incised to expose kidney capsule (*n* = 5) with small incision for implanting ILCC. The abdominal cavity and overlying skin were sutured back and animals were allowed to recover for 2 weeks. Other groups of mice (*n* = 5) were taken as diabetic control. FBG was monitored weekly till four weeks.

### 2.13. Histological and Immunohistochemistry Assessment of ILCC after Graft Excision

Transplanted ILCC from kidney graft were excised 4 weeks after surgery and were histologically examined. Eight micron thick sections were used, deparaffinized, and rehydrated using varying alcohol grades of 100% for 5 min and 90%, 70%, 50%, and distilled water for 1 min each. Each section was then incubated in blocking solution at room temperature for 1-2 hours. Primary antibodies for insulin, c-peptide, and glucagon (for details, see Table 1) were incubated overnight at 4°C. After washing three times with 0.1 M PBS for 10 min, FITC conjugated secondary antibodies were incubated for 1 h in 0.1 M PBS at room temperature. Nuclei were visualized with 4-9-6-9 diamidino-2-phenylindole (DAPI) and sections were mounted with VECTASHIELD antifade mountant. Images were recorded using confocal microscope (LSM710, Carl Zeiss, Germany).

### 2.14. Statistical Analysis

The data are given as mean ± SEM. The significance of difference was evaluated by the paired Student's *t*-test. When more than one group was compared with one control, significance was evaluated according to one-way analysis of variance (ANOVA).

## 3. Results

### 3.1. Isolation of Compound from Ethyl Acetate Fraction Characterization: Identified as Swertisin

**Figure 2:**
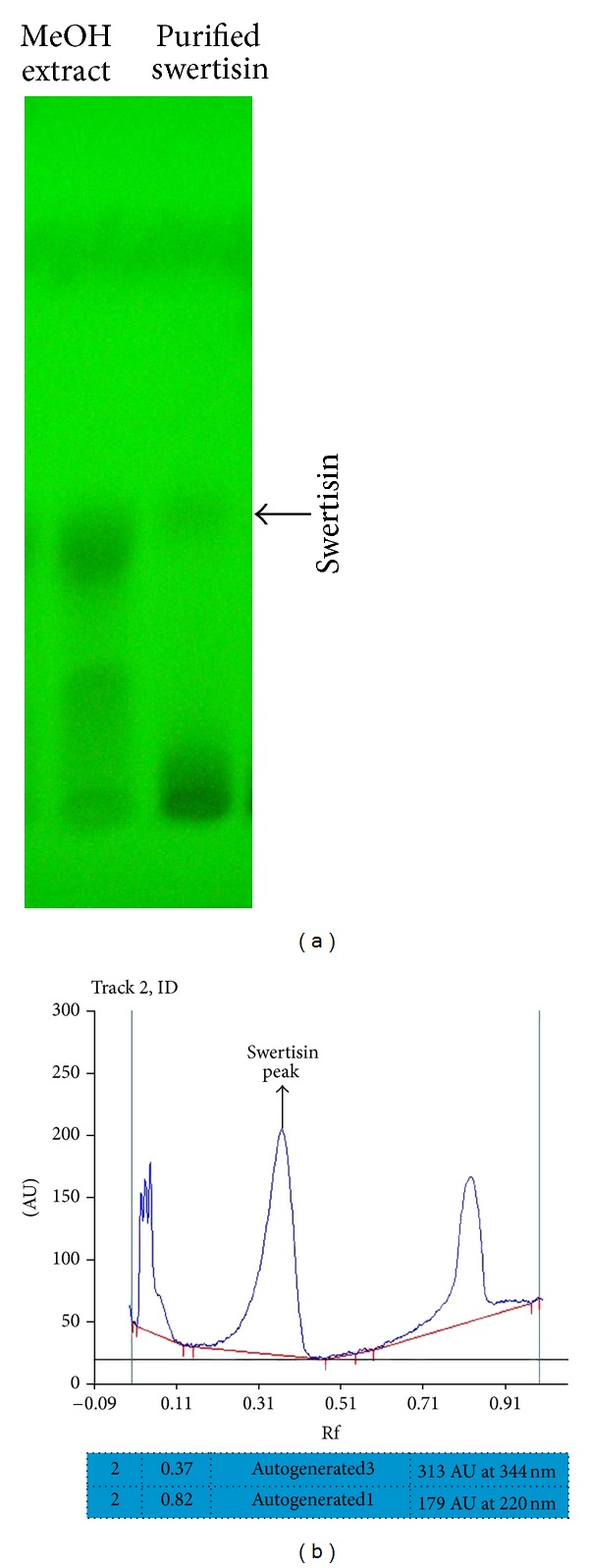
TLC and HPTLC profile of swertisin compound: (a) represents TLC plate scan at UV-265 nm showing methanolic extract and purified swertisin compound profile as a single band at Rf 0.32 (arrow marked), and (b) represents HPTLC curve showing single peak of swertisin at Rf 0.32 and UV-344 nm wavelength.

**Figure 3:**
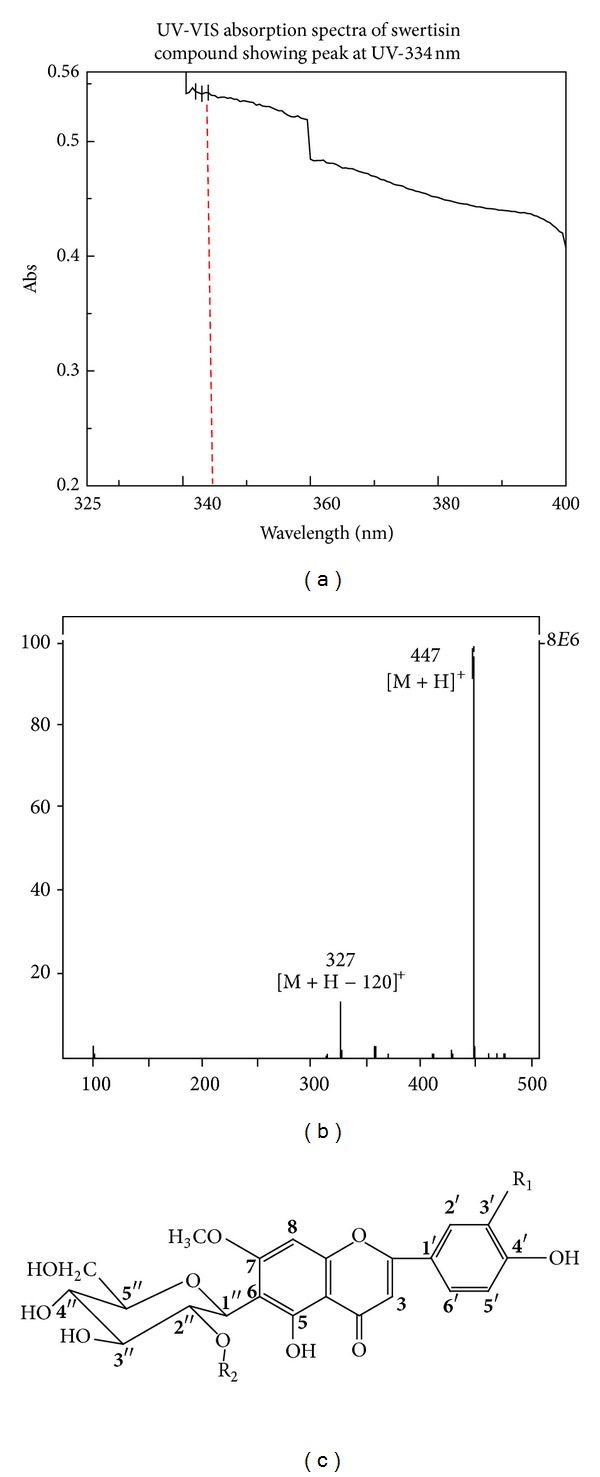
UV-Vis and mass fragmentation pattern of swertisin compound: (a) represents UV-Vis absorption spectra of swertisin compound. a base peak at height of 0.54 at 344 nm was seen purified swertisin (red line marked). (b) represents mass fragmentation curve showing two single peak 447 [M + H]^+^ representing swertisin compound and 327 [M + H − 120 u] release of Glycosyl ring. (c) shows structure of swertisin based on mass fragmentation pattern.

The compound isolated from ethyl acetate fraction of *E. littorale* was confirmed as a single peak at 344 nm in UV-Vis spectrum analysis (Figure 2) and a single spot at Rf 0.72 of HPTLC plate again at *λ*_max⁡_ of 344 nm when scanned on CAMAG TLC Scanner-3 system (Figures 2(a) and 2(b)). These results indicated the presence of swertisin compound as reported earlier by Patel and Mishra and Colombo et al. [18, 20]. Mass fragmentation pattern of this compound was also compared with earlier references and found to be identical to swertisin [20]. Our results showed a base peak of *m/z* 447 [M + H] which corresponded to molecular weight of swertisin in positive ionization mode. Further more, a peak of M + H − 120 was also obtained which indicated a loss of 120 u a characteristic peak of C-glycosyl flavonoids as mentioned by Colombo et al. (Figure 3(b)) [20]. The compound was found to be soluble in solvents like DMSO and dioxane, while sparingly soluble in methanol and insoluble in water. All the above results shown by us are in accordance of swertisin reported data with structure shown in Figure 3(c).

### 3.2. Morphologic and Phenotypic Characteristics of Undifferentiated NIH3T3 Cells

**Figure 4:**
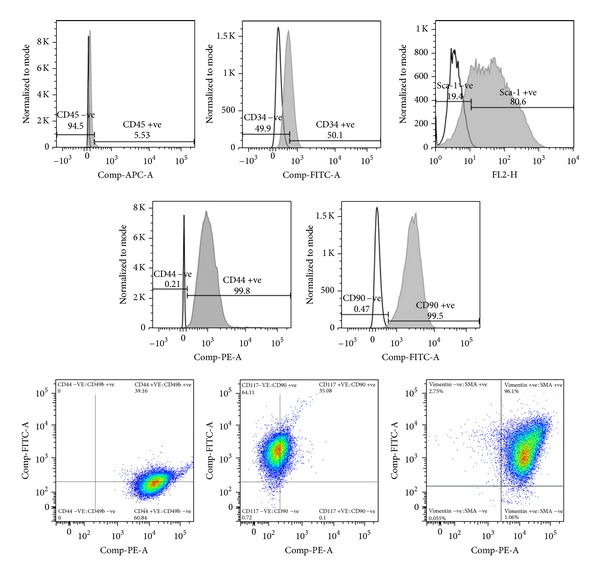
FACS dot plots of various stem cell markers in NIH3T3 cells. The figure depict histogram and dot plots of flow cytometry data analyzed for (1) CD34, (2) CD44, (3) CD45, (4) CD49b, (5) CD117, (6) CD90, (7) Sca-1, (8) Vimentin, (9) SMA.

**Figure 5:**
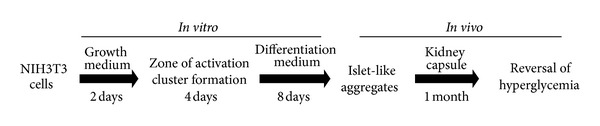
Schematic protocol for NIH3T3 cells normal growth and their differentiation to functional islet-like cells clusters (ILCC). The protocol involves 8-day *in vitro* differentiation into islet-like cell clusters that mature *in vivo* into functional organelle, following transplantation under the kidney capsules of BALB/c mice.

**Figure 6:**
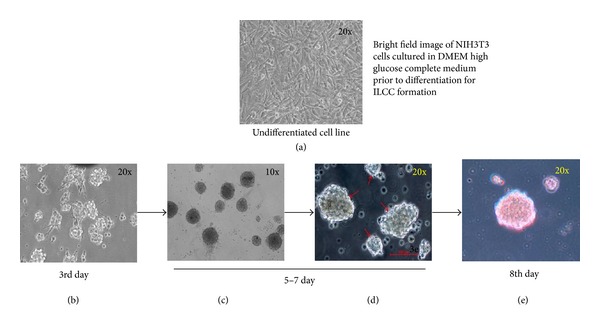
Schematic representation of event occurring in swertisin-induced ILCC formation. (a) represents basic fibroblastic morphology of NIH3T3 cells cultured in complete medium. (b)–(d) depicts event occurring during swertisin-induced differentiation process from the 3rd day to the 8th day. (e) represents DTZ staining in differentiated ILCC for primary confirmation of beta cell differentiation.

At full confluence, NIH3T3 cells became homogenous and attained spindle shape, fibroblast-like morphology, and formed a monolayer (Figure 6(a)). Flow-cytometric data showed positive peaks for mesenchymal stem cell markers CD44, c-kit (CD117), CD49b, CD90, Sca-1, Vimentin, SMA, and a hematopoietic stem cell marker CD34 but negative for CD45 (Figure 4). We observed 99.8% cells positive for CD44, 99.5% cells positive for CD90, 80.56% cells positive for Sca-1, 50.1% cells positive for CD34, and *∼*5% cells positive for CD45. These cells also showed 39% staining for positive for both CD44 and 49b and *∼*35% cells showed dual staining for CD90 and 117 (ckit). Further more 96% cells stained positive for both Vimentin and smooth muscle actin. This result indicated stem/progenitor-like features in NIH3T3 cells similar to multipotent stem cells [21]. 

### 3.3. Swertisin Effectively Potentiates Islet-Cell Differentiation from NIH3T3 Cells

**Figure 7:**
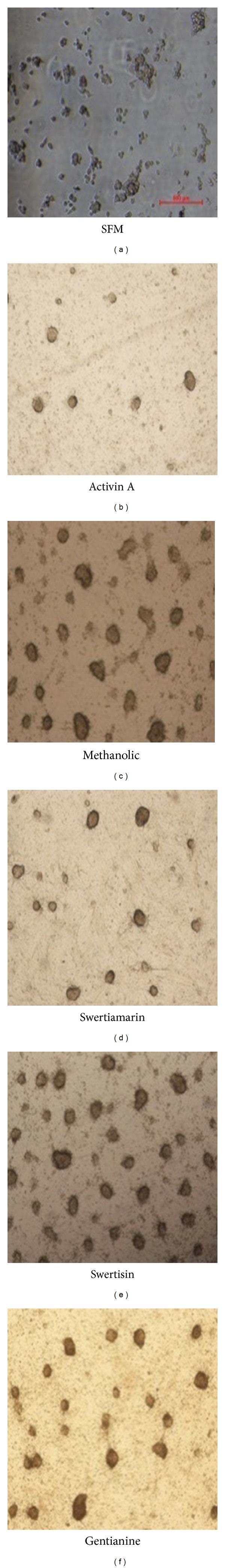
Pictorial representation of ILCC generation from control, activin A and various other *E. littorale *compounds: (a)–(f) demonstrate comparative pictorial representation of ILCC generation from NIH3T3 cells using SFM control, activin A and various EL compounds. (e) indicates maximum ILCC generation with swertisin induction.

In order to identify most potent islet differentiating agent among various isolated compounds from *E. littorale*, a comparative islet differentiation was carried out with various compounds using NIH3T3 cells as extra pancreatic progenitor cells for 8 days along with positive islet differentiation inducer agent activin A. After 8-day induction, cluster formation was observed microscopically where swertisin-induced group showed maximum zone of activation and cell aggregation followed by gentianine (Figure 7). ILCC yield and morphological features like average size and area were quantified. Swertisin-induced NIH3T3 cells showed the highest yield of ILCC with significant increase of 3.9-fold compared to SFM alone and activin A induced differentiation group (Figure 15). On calculating average area of ILCC generated amongst all groups, no significant difference was observed in all tested compounds, compared to control, indicating that clusters generated were more or less of similar area (Figure 16). 

More pertinently, swertisin and gentianine both showed significantly higher number of ILCC categorized in two groups based on size, ranging from 150 to 300 *µ*m (Figure 17) and from 300 to 3000 *µ*m (Figure 18). On the other hand, activin A generated more numbers of ILCC ranging between 150 to 300 *µ*m and less between 300 to 3000 *µ*m. An increase of approximately 9.3-fold bigger size ILCC from swertisin compared to activin A eventually demonstrated generation of mature ILCC. These results represent the mean of three independent observations and differences were found to be statistically significant (*P* < 0.01  and  0.001). To further test the mode of islet differentiation between control SFM, activin A, and swertisin, microscopic observations were recorded in time-dependent manner and confirmed for insulin biogenesis. After induction with swertisin, the morphology of NIH3T3 cell gradually changed from a fibroblast-like shape to round cells within 2 days and large number of cells formed tight clusters (Figures 6(a), 6(b), and 6(c)). The differentiated NIH3T3 cells showed intense zone of activity and clustering in just 4 days with swertisin induction (Figures 6(c) and 6(d)). These clusters turned up to mature ILCC within 8 days and floated into the medium resembling to islet-like miniorganelles.

### 3.4. DTZ and C-Peptide Staining Depicts Islet Differentiation in NIH3T3 ILCC

**Figure 8:**
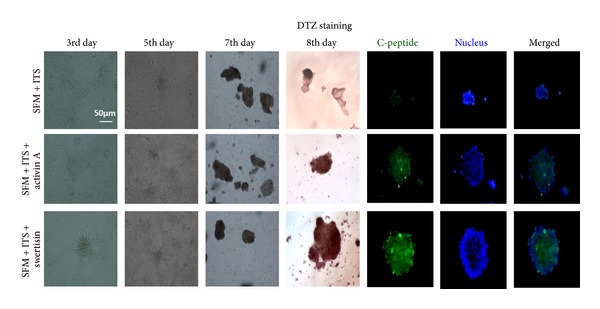
Comparative montage of morphological events occurring in 3–8 day time duration. The figure represents day-wise representation of morphological changes and differentiation event occurring with SFM, activin A and swertisin-mediated ILCC. The figure also shows DTZ staining in differentiated ILCC for primary confirmation of beta cell differentiation and C-peptide staining representing presence of insulin biogenesis and insulin granules.

We examined the clusters generated in each group, for insulin-producing cells, and stained them with zinc chelating agent DTZ which is a zinc-binding substance. Pancreatic islets are known to stain crimson red in color after DTZ incubation. Swertisin-induced NIH3T3 ILCC were distinctly stained crimson red by DTZ indicating a sequestration of zinc along with insulin granules (Figure 8). To further examine the new insulin biogenesis, we carried out C-peptide staining. C-peptide is a 3-4 kDa peptide released from insulin molecules within the beta cells. In accordance to our expectations, when swertisin-induced differentiated clusters were tested for C-peptide, along with SFM and activin A group, we found immense positive cytoplasmic staining of mouse C-peptide in swertisin generated ILCC whereas activin A mediated ILCC showed less staining with no staining in control SFM clusters (Figure 8, also see Figure SP-2 available online at http://dx.doi.org/10.1155/2013/280392).

### 3.5. Confocal Imaging Demonstrates Swertisin-Mediated ILCC Possess Proper Islet Architecture

**Figure 9:**
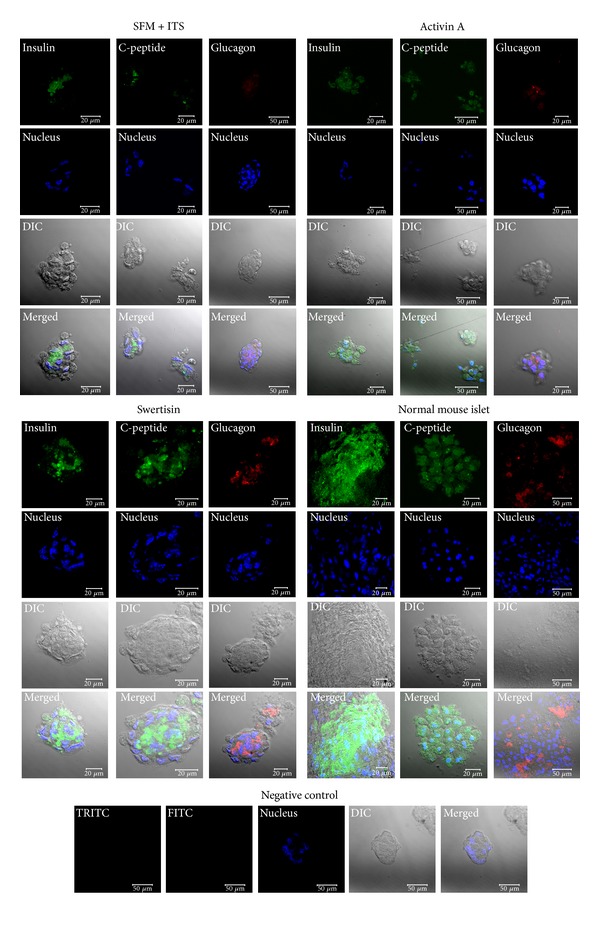
Confocal images of various islet markers in SFM + ITS, activin A, and swertisin induced ILCC. The figure depicts intense positive staining for insulin (green color), C-peptide (Green color), and glucagon (red color). Nucleus of all the clusters was stained with DAPI shown in blue color.

To monitor the presence of various islet hormones and differentiation markers, immunocytochemical staining was performed in cell clusters generated with SFM + ITS, activin A, and swertisin induction and stained for PDX-1, insulin, C-peptide, and glucagon proteins. PDX-1 is considered as an important marker for initiation of islet differentiation. Pancreatic progenitor cells are known to be highly PDX-1 expressing. Many groups have also shown that ectopic expression of PDX-1 lead to islet cell differentiation [22]; moreover, it is also reported that even mature beta cells do express basal level of PDX-1 protein [23]. In our study, we observed that undifferentiated NIH3T3 cells were slightly PDX-1 positive (Figure 9) but negative for C-peptide staining, depicting potential to form ILCC similar to pancreatic progenitors. Upon differentiation with activin A, we observed NIH3T3 ILCC to show both insulin and C-peptide staining. Similarly, swertisin-induced ILCC also stained intensely positive for C-peptide, insulin, somatostatin, and glucagon hormones (Figure 9). 

### 3.6. RT-PCR/Q-PCR Data Revealed Potent Islet Differentiation by Swertisin Induction

**Figure 10:**
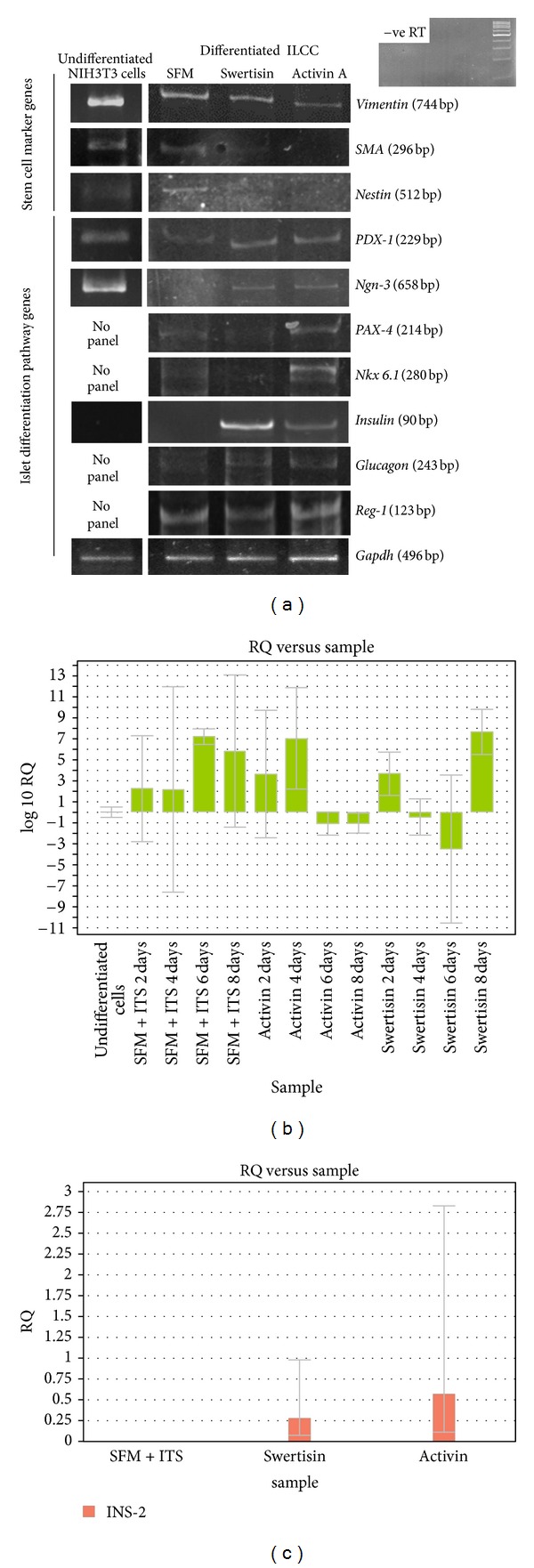
RT-PCR and Q-PCR expression data of various stem/progenitor and islet differentiation pathway genes. (a) shows mRNA expression of various stem/progenitor genes and key islet differentiation pathway transcription factor genes harvested at 10 days of differentiation protocol in SFM alone, activin A, and swertisin-mediated ILCC. (b) shows comparative PDX-1 transcript expression in differentiated clusters harvested at different timepoints. (c) represents insulin mRNA transcript levels in SFM + ITS, activin A, and swertisin-induced clusters at the 8th day.

Using reverse transcriptase PCR and Q-PCR, mRNA expression of islet differentiation pathway genes and mature islets were quantified. In our study, mRNA transcripts were not detected in the undifferentiated NIH3T3 cells neither for pancreatic endocrine development genes nor for islet specific genes. However, undifferentiated NIH3T3 cells were found to be strongly expressing transcripts of stem/progenitor related genes like Nestin, Vimentin and smooth muscle actin (SMA). Upon differentiation, we found significant elevated expression of both differentiation pathway transcription factors like Nestin, PDX-1, Ngn-3, Pax-4, Nkx 6.1, and Reg-1 and endocrine genes like insulin and glucagon at the end in swertisin induced ILCC with significant reduction in expression for stem cell markers like Nestin, Vimentin, and SMA genes that was also noted (Figure 10(a)). The most peculiar thing about swertisin induction is that the flavonoid not only potentiates islet differentiation pathway, but also accelerates the pace of differentiation. This phenomenon is more obvious with significant reduction in Nestin, Vimentin, and SMA transcript and absence of Pax-4 and Nkx 6.1 expression in swertisin-induced clusters while the same is elevated in SFM alone and activin A induced ILCC even at 8th day. Expression of endogenous control gene Gapdh confirmed the uniformity of the experimental system and acted as input template control.

Also the Q-PCR data demonstrated increase in PDX-1 transcripts at early timepoint and goes down again as the process of differentiation progress in time-dependent manner. Our results clearly showed that a steady increase in PDX-1 transcript in SFM with ITS control group initiated from day 2 and sustained till day 8, whereas when treated with activin A, the mRNA transcript first increased for the first 4 days and then declined significantly by day 6 and continued till day 8. Swertisin also showed increase in PDX-1 transcript right away from day 2 but declined by day 4 and 6 and further increased on day 8. The data showed early onset of differentiation in swertisin group with low levels in late differentiation phase hence not observed at day 10 or so (Figure 10(b)). We also performed real-time quantitative PCR for mouse insulin gene (INS-2 gene). Our data showed that both activin A and swertisin showed increased insulin transcript number while SFM + ITS fails to show the same on the 8th day (Figure 10(c)).

### 3.7. Immunoblot Confirmed Islet Differentiation Pathway Facilitation by Swertisin Induction

**Figure 11:**
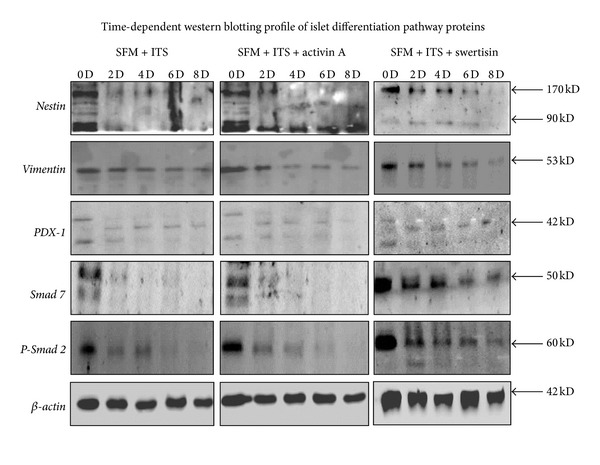
Time-dependent western blot study of various stem/progenitor and islet differentiation pathway protein. The figure shows time-dependent expression of various stem/progenitor markers and key islet differentiation pathway transcription factor during differentiation protocol ranging from day 0 to day 8.

Protein immunoblotting was performed to monitor the initiation and fate of differentiation signaling pathway operated with SFM alone, activin A, and swertisin induction. Major transcription factors like Nestin, PDX-1, phospho-Smad-2, and Smad-7 (key signaling modulator for endocrine fate) for Ngn-3 up-regulation (a master regulator protein for islets) were analyzed in a time dependent manner from day zero to day eight. In this kinetic study, we observed that SFM-induced ILCC fail to downregulate stem/progenitor marker vimentin, resulting in no differentiation, whereas both activin A and swertisin showed decrease in vimentin from the 4th day onwards (Figure 11 and Figure SP-1). Compared to activin A, swertisin clusters showed more steep decrease in vimentin protein, which was completely lost by the 8th day. 

Further, SFM alone clusters failed to show upregulation of Nestin, and PDX-1 proteins, which are crucial for initiation of differentiation (Figure 11), but the same was found to be elevated in both activin A and swertisin, and more prominently in swertisin-induced ILCC. Activin A induced ILCC showed an increase in Nestin protein expression (cleaved form 90 kDa) starting from day 2 which continued even till day 8 (Figure 11) suggesting a slower pace in differentiation process. On the contrary, swertisin generated ILCC showed upregulation of both forms of Nestin 170 kDa and 90 kDa which peaked at the 4th day, decreased significantly by the 6th day and completely lost by the 8th day, representing complete and proper differentiation of NIH3T3 cells with swertisin induction. Apart from above observations, swertisin mediated clusters also showed high expression of PDX-1 protein within 2 days and persisted even till the 8th day, whereas both SFM alone and activin A showed no similar trends (Figure 11). 

Smad proteins are known to play a role in islet differentiation mediated by activin A. To purely understand the fate of islet differentiation by swertisin we also targeted Smad signaling by monitoring Smad2 and 7 proteins. In a day-wise study, we found that swertisin clusters showed dramatic decrease in Smad7 expression by day 2 with continuous increase in Smad2 phosphorylation till day 8, whereas activin A showed less Smad2 phosphorylation inspite of steep decrease in Smad7 expression (Figure 11). A similar pattern was seen with SFM alone group too, which failed to phosphorylate Smad2. It is important to note that Smad protein phosphorylation in swertisin clusters clearly demonstrates more immense potential for islet differentiation than activin A.

### 3.8. *In Vitro* Insulin/C-Peptide Release Assay Indicated Glucose Responsiveness of Swertisin-Generated ILCC

**Figure 12:**
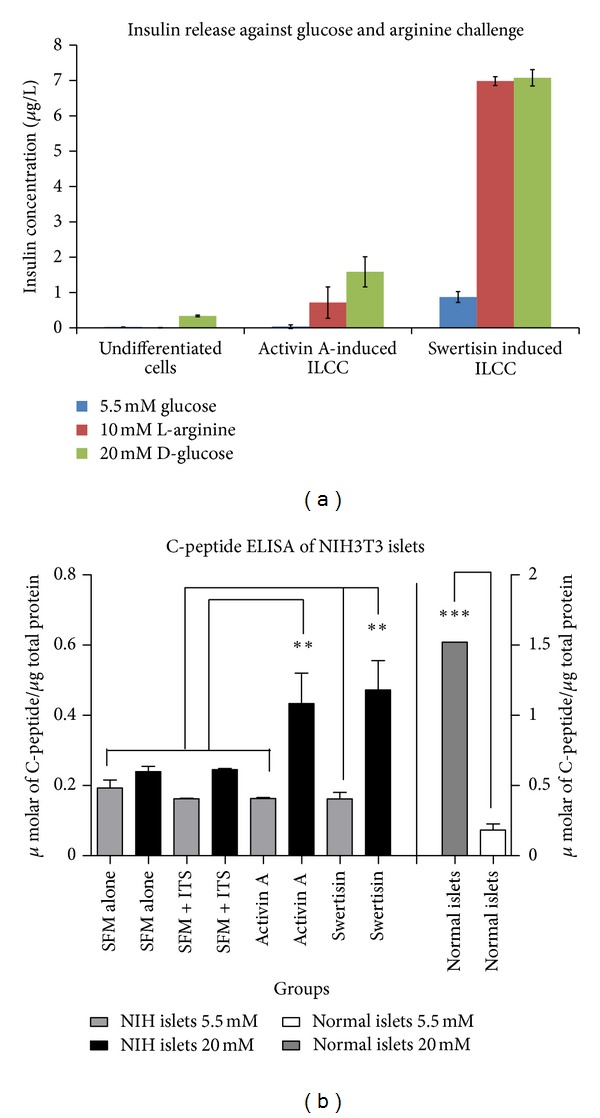
Glucose responsive insulin and C-peptide release assay. (a) represents glucose responsive insulin release from SFM, activin A and swertisin-mediated ILCC with increasing glucose and L-arginine concentration. Results are expressed as mean ± SEM of three independent observations, *N* = 3. (b) represents glucose responsive C-peptide release from SFM, SFM + ITS, activin A and swertisin-mediated ILCC. Results are expressed as mean ± SEM of three independent observations, *N* = 3.

To further check the functional maturity of swertisin-induced ILCC, *in vitro* glucose challenge was performed to monitor them for increasing amounts of insulin or C-peptide in a glucose-concentration-dependent fashion (Figure 12(a)). The mean insulin secretion by swertisin-mediated clusters (100) was 0.87 ± 0.022 *µ*g/mL in response to 5.5 mM glucose, 6.98 ± 0.42 *µ*g/mL in response to 10 mM L-Arginine, and 7.07 ± 0.23**** *µ*g/mL in response to 20 mM glucose concentration, which is significantly higher than SFM alone (0.025 ± 0.009 *µ*g/mL in 5.5 mM, 0.004 ± 0.052 *µ*g/mL in response to 10 mM L-Arginine, and 0.337 ± 0.15 *µ*g/mL in 20 mM glucose concentration) and activin A (0.035 ± 0.003 *µ*g/mL in 5.5 mM, 0.716 ± 0.442 *µ*g/mL in response to 10 mM L-Arginine and 1.584 ± 0.12 *µ*g/mL in 20 mM glucose concentration). Swertisin-differentiated ILCC showed 20.9, and 4.46 fold increase in insulin release compared to SFM and activin A induced ILCC, respectively, upon 20 mM glucose challenge. The result represents mean of three observations and differences were found to be statistically significant (*P* < 0.01). We also performed C-peptide release from the same ILCC in order to confirm new insulin synthesis as there is a possibility of uptake of insulin by endocytosis. By C-peptide ELISA we confirmed the presence of new insulin biogenesis as C-peptide release from both swertisin and activin A and was found to be significantly higher at 20 mM glucose induction compared to 5.5 mM induced clusters similar to normal mouse islets. When we compared SFM alone and SFM + ITS induced clusters, we could not find any significant release in C-peptide even upon high glucose challenge (Figure 12(b)). 

### 3.9. STZ-Mediated Diabetes Induction and Islet Transplantation in Renal Capsule

**Figure 13:**
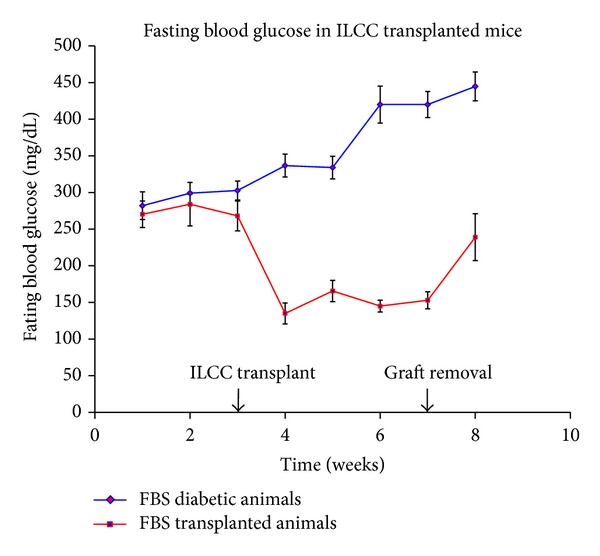
FBG in BALB/c mice after transplantation. The figure represents fasting blood glucose in time-dependent manner from swertisin-mediated transplanted ILCC and diabetic control mice. Glucose was monitored for 7 weeks, and 1 week after graft removal. Results are expressed as mean ± SEM of 5 transplanted animals.

To check the functionality of swertisin-induced NIH3T3 ILCC for insulin cell functionality *in vivo*, STZ-induced diabetic mice model was used. Multiple dose of STZ in BALB/c mice produced effective TD1 mice which greatly elevated blood glucose levels (Figure 13). The efficacy of glucose lowering tendency from swertisin-induced ILCC was monitored from transplanted renal grafts. All the mice receiving renal grafts showed significant reduction in FBG within one week of transplantation (Figure 13).

### 3.10. H&E Staining and Immunohistochemistry of Transplanted Renal Grafts Reconfirm *In Vivo* Functionality of Swertisin-Induced ILCC

**Figure 14:**
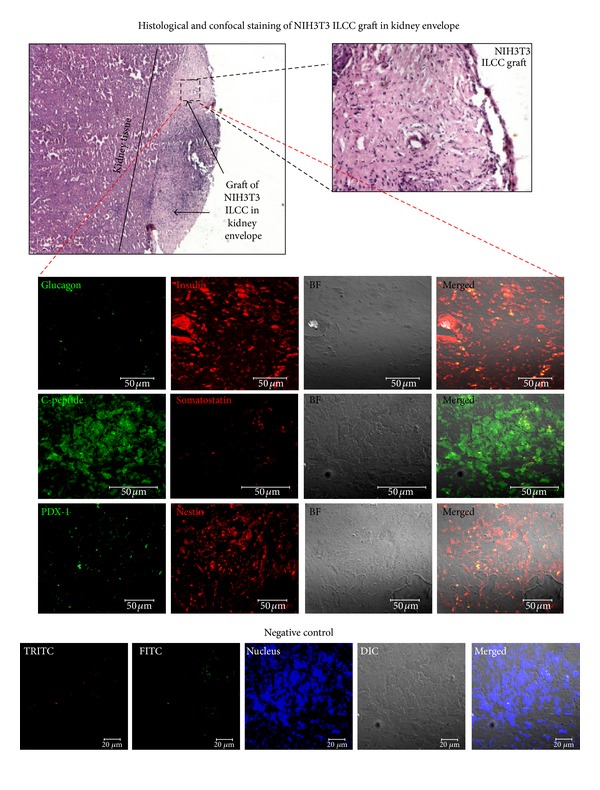
Histological and immunochemical staining of islet hormones in kidney-ILCC grafts. Top first two figure represents a histological observation of kidney graft from transplanted swertisin-mediated ILCC. Islet specific makers were also observed in these grafts, which show insulin (red), C-peptide (green), somatostatin (red), Pdx-1 (green), and Nestin (red).

**Figure 15:**
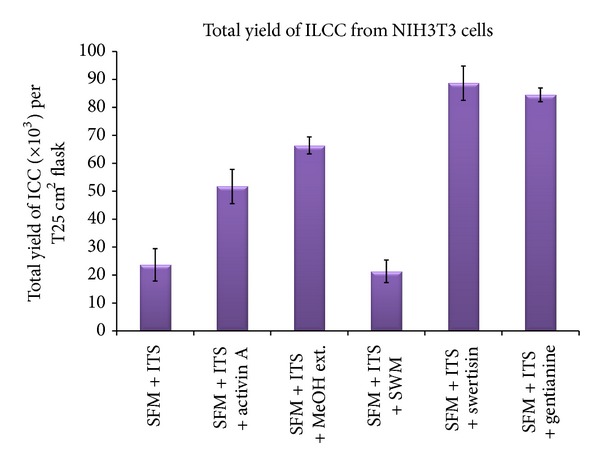
The figure depicts total yield of ILCC where swertisin showed significantly higher yield compared to SFM and activin A group.

**Figure 16:**
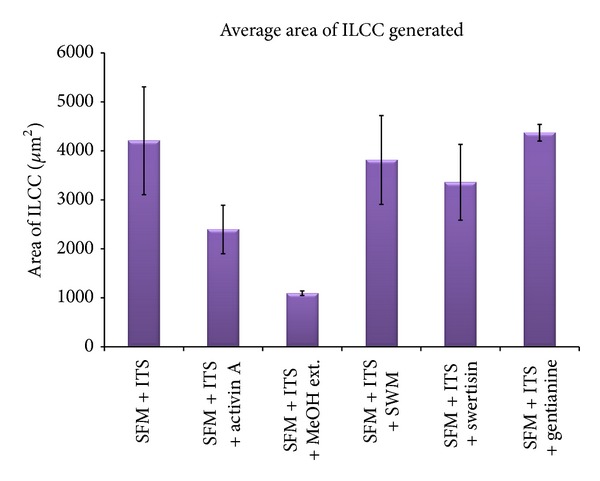
The figure shows comparative data of average area of ILCC generated with various compounds.

**Figure 17:**
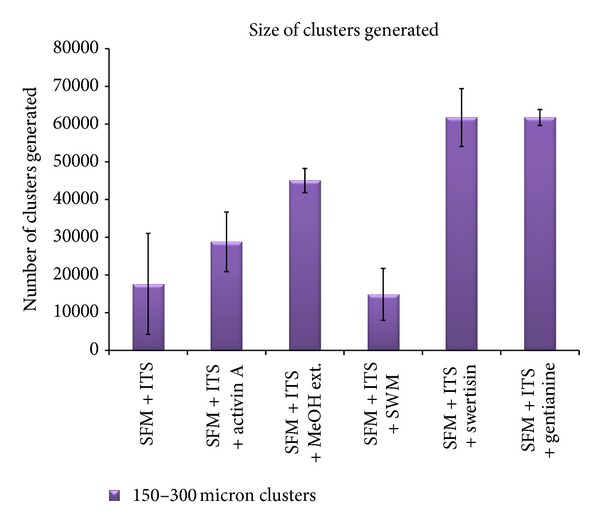
The figure represents size of ILCC clusters generated between range of 150–300.

**Figure 18:**
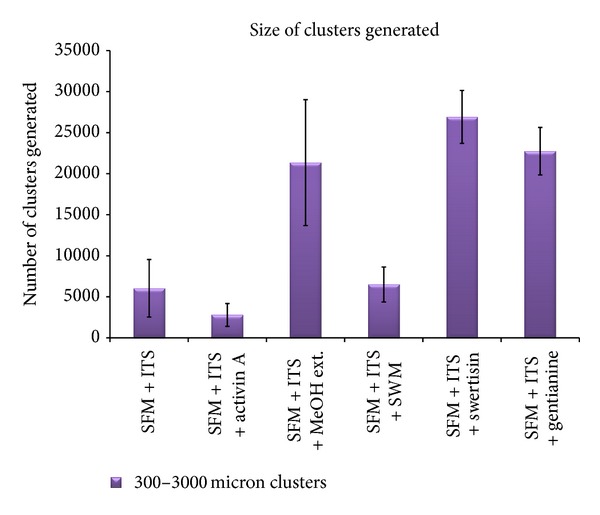
The figure represents size of ILCC clusters generated between range of 300–3000 *μ*m.

To demonstrate the viability and functional integrity of transplanted islet grafts *in vivo* after stabilization period of four weeks, renal capsule having transplanted ILCC grafts was excised, sectioned, and histologically examined for intact islet architecture using H&E stain. Deep eosin cytoplasmic with less nuclear hematoxylin stain showed compact islet architecture. More of eosin staining resembles the graft staining similar to pancreatic islet section stain. Presence of C-peptide, insulin, glucagon, and somatostatin using immunohistochemistry was also assessed. Intense cytoplasmic staining for all the four hormones in excised tissue showed that transplanted ILCC graft was not only viable but also functional during transplantation period inside mouse kidney, whereas kidney cells did not stain for any islet hormone (Figure 14).

## 4. Discussion

The present study reframes a new arena to screen and identify the islet neogenic potential of an important compound from *E. littorale* plant to develop cheaper but readily available drug solution for diabetic patients. The literature in recent years has been repleted with a spectrum of experimental evidences that demonstrates the possibility of generating insulin producing cells from embryonic as well as adult stem cells with numerous growth factors [24–26]. Despite their promising potential for differentiation, it is still difficult to obtain sufficient islets for autologous islet transplantation [1]. One of the important obstacles is the lack of potent differentiating agents which can yield enormous amount of islet cells from starting pool of stem cells. Few differentiating agents like activin A, a member of TGF-*β* family, which converts embryonic stem cells to islet cells [27], glucagon like peptide-1 and keratinocyte growth factor are reported with tissue specific stem cell differentiation into islets [28]. Nicotinamide and exendin-4 are more such compounds which show islet neogenesis by inhibiting poly ADP-ribose synthesis [29], stimulating both *β* cell replication and neogenesis from ductal progenitor cells and inhibiting apoptosis of *β* cell [30, 31]. Above all the major impediment to it use as a therapeutic drug is the high cost which makes it unreachable to patients. To overcome this limitation, we screened islet differentiation potential of bioactive agents from medicinal herbal plants. Identification of such potent herbal active ingredient may revolutionize the therapeutic approach for achieving enormous islet yield for effective treatment of diabetic patients. 

Numerous plants and their products have been demonstrated for antidiabetic activity in both cellular and animal models [6–9]. The majority of them were targeted to emphasize the ameliorating effect of hyperglycemia either by increasing insulin secretion or by sensitizing downstream signaling [32, 33]. Fewer reports underwent extensive research to monitor the status of diabetic stressed beta cells within the pancreas upon such herbal treatments and mechanisms of plant products mediated beta cell replenishment. Few *in vitro* studies highlighted the role of certain herbal products in stem cell reprogramming into functional insulin-producing cells. In 2003, Kojima and Umezawa, have reported one such compound named conophylline showing AR42J cells conversion into insulin positive cells [10]. Another group, Ansarullah et al., has recently investigated the *in vivo* islet replenishing property of *Oreocnide integrifolia* in pancreatectomized mice [34]. 

In similar directions, the author's group has also reported islet differentiating activity from *E. littorale* plant earlier in 2010 [17]. Understanding and identifying the most potent islet neogenic active principle of this plant serves the aim of this study, which is the most essential criteria for developing the molecule as an islet cell replacement and therapeutic agent. In comparative screening for various extracts and isolated purified compounds from *E. littorale* methanolic extract, we observed islet cluster formation activity using NIH3T3 cells. It is significant to note that NIH3T3 cell differentiation to certain cell types have previously been reported by few groups, except islet differentiation property [35, 36]. *E. littorale* compounds induced effective differentiation of NIH3T3 cells similar to activin A. The rational for using activin A as positive control was due to its reported ES cell to islet differentiation activity and NIH3T3 cells are also from embryonic in origin [27]. The screening of all other compounds in this fashion provided the most potent inducing agent which possesses the highest islet neogenic potential with the generation of a a large number of both smaller and bigger sized ILCC. This primary screening provided us a basis to identify this compound, which was further characterized for its structure and molecular differentiation pathway leading to insulin-producing cells.

For structure identification of an unknown compound, it is mandatory to first evaluate its primary characteristic-like UV absorption spectra, TLC pattern, HPTLC curve profile, and so forth [37]. Colombo et al. have previously reported UV spectrum and HPTLC plots for various compounds from *E. littorale* [38]. We examined and compared our results with another report by Sawant et al. and found that our compound of interest shows identical Rf and absorption maxima values with swertisin as reported by these two groups [39]. Structural identification was further performed by ESI MS/MS spectroscopy. Mass fragmentation pattern of a base peak of *m/z* 447 [M + H] corresponded to swertisin structure, similar to earlier reports by Colombo et al. and Sawant et al. [38, 39]. Therefore, we confirmed that the compound we isolated is indeed swertisin which has enormous islet neogenic potential.

One of the most logical modalities to confirm beta-cell differentiation is to monitor for zinc chelating agent dithizone (DTZ) staining in newly generated clusters [40]. Differentiation of NIH3T3 cells into islet cells in 8-day protocol generated large and mature clusters. Insulin production was confirmed by staining with DTZ. Some investigators argue that part of insulin detected may have been derived from insulin added to the culture media in certain protocols or insulin present in serum [41]. Therefore, it becomes essential to demonstrate new biogenesis of insulin molecule by C-peptide staining in newly differentiated clusters. Detection of C-peptide in newly generated clusters confirmed that insulin presence was the result of endogenous synthesis. Presence of insulin or C-peptide is not sufficient enough to regulate glucose, but presence of glucagon and somatostatin is also needed to maintain the homeostasis. Hence, islet transplant becomes a better strategy than that of *β* cells alone [42]. We observed the presence of other islet hormones in ILCCs generated by swertisin induction. The expression of glucagon and somatostatin in the ILCCs indicates that swertisin not only produces insulin positive cells, but it also facilitates the differentiation of glucagon and somatostatin positive cells to provide more or less a complete islet phenotype with mature islet architecture.

Some groups have reported that stem cell differentiation into islet cells follows embryonic ontology and their gene expression of differentiated clusters should be similar to that of pancreatic endocrine tissue [43]. Zhang et al. [44] reported that nestin positive precursor cells possess instructive signals that govern islet differentiation pathway. Ngn-3 is known to be the key master regulator of endocrine cell fate; initiation of islet differentiation signaling occurs by early induction of Ngn-3 protein [45]. The present study showed early induction of differentiation pathway genes like Nestin, Pdx-1, Ngn-3, Pax-4, Nkx 6.1, and Reg-1. The expression of insulin, Pdx1, Reg-1, and Nkx 6.1 is considered to be a specific functional characteristic of mature *β* cells and has previously been reported only *in vivo* [46].

Differentiation into insulin producing endocrine cells is induced by a cascade of signaling proteins controlled by several transcription factors such as Nestin, PDX-l, Neurogenin-3, and Smad-2 [47]. Abundant expression for Smad2 and Smad4 with low expression of Smad1, Smad3, and Smad7 was earlier reported by Zhang et al. [48] in developing pancreas. This Study showed that swertisin upregulates Ngn-3 in early days and goes down thereafter (data not shown). This in turn showed downstream repression of Smad7 while over expression of Smad2 leading to mature islet differentiation similar to activin A [47]. All these results were found to be in accordance with earlier reports by numerous groups [49]. Evidence has also been provided that swertisin induction showed significant high release of insulin upon glucose stimulation in a dose-dependent manner, suggesting that these cells possess the major functional capabilities of *β* cells, namely, insulin release in response to changes in extracellular glucose concentrations and the presence of insulin-containing secretory granules [50].

Progress towards the goal of direct assessment of graft integrity, viability, and function is mandatory for therapeutic application. Observing animal response for blood glucose under diabetic condition is a real functional challenge for differentiated clusters. Many studies previously demonstrated reversal of hyperglycemia upon transplantation of differentiated ILCC's under kidney envelope [29]. Kidney being a highly vascular organ supports survival and ideal niche for the functional maturity of newly generated ILCC. Glucose lowering effect of transplanted NIH3T3 ILCC under diabetic BALB/c mice kidney capsules confirmed that swertisin mediated clusters do possess functional maturity and are able to combat diabetic conditions. The molecular proof of this can be visualized by the fact that kidney graft isolated from transplanted animals showed the presence of viable islet architecture having immunopositivity for C-peptide, insulin, glucagon, and somatostatin along with basal expression of nestin and PDX-1. Furthermore, a fasting blood glucose response showed that the blood glucose levels of transplanted diabetic mice exhibited similar kinetics to those of normal control mice [29], representing a recovery of insulin secretory ability in the swertisin-mediated NIH3T3 ILCC transplanted mice. Taken together, these results suggest that these induced cells have a similar function to normal physiological islets both *in vitro *and *in vivo.*

Therefore, in conclusion, we state that swertisin is a novel molecule which can serve as an efficient differentiating agent generating islet-like cell types, with enormous yield and mature functional status. Despite these encouraging observations, in the authors' opinion, the issue of generating islet-cell mass *in vitro* and surviving *in vivo* using less expensive and readily available differentiating agents still remains a challenge unless we attempt to explore more medicinal herbal products with potential stem cell conversion for miraculous therapeutic activities like islet neogenesis.

## Supplementary Material

Figure SP-1: represents densitometric quantification of vimentin protein expression and p-Smad-2 protein expression in 0-8 day time course in various NIH3T3 differentiation islet like clusters groups. Data is represented as Mean Band Density normalized to endogenous control beta actin.
Figure SP-2: representing comparative flowcytometery quantification data of islet cell markers like insulin, c-peptide, glucagon from differentiated NIH3T3 islet like clusters and normal mouse islet cells. All three groups control SFM, Activin-A and Swertisin are compared to normal mouse islets cells.

## Figures and Tables

**Table 1:** List of antibodies used for flow cytometry, immunohistochemistry, and western blotting.

Name of antibody	Company	Target against species	Mono/polyclonal	Molecular weight (kDa)	Source	Dilution for WB	Dilution for ICC	Dilution for flow cytometry
Beta actin	Thermo scientific	Hu, Rt, Mu	Mono	42	Mouse	1 : 5000	NA	NA
p-Smad-2	Cell signalling	Rt, Mu	Poly	60	Rabbit	1 : 1000	NA	NA
Smad-7	R & D systems	Rt, Mu	Mono	50	Mouse	1 : 1000	1 : 100	NA
Nestin	Sigma-Aldrich	Hu, Rt, Mu	Mono	177	Rabbit	1 : 1000	1 : 250	NA
Pdx1	Cell signaling	Hu, Rt, Mu	Mono	42	Rabbit	1 : 1000	1 : 250	NA
Vimentin	Sigma	Hu, Rt, Mu	Mono	53	Mouse	1 : 1000	1/250	NA
CD90-FITC	BD	Hu, Mu	Mono	—	Mouse	—	1 : 100	1 : 10
CD34-FITC	BD	Hu, Mu	Mono	—	Mouse	—	1 : 100	1 : 10
CD45-APC	BD	Hu, Mu	Mono	—	Mouse	—	1 : 100	1 : 10
CD49b-FITC	BD	Hu, Rt, Mu	Mono	—	Mouse	—	1 : 100	1 : 10
CD117-PE	BD	Hu, Rt, Mu	Mono	—	Mouse	—	1 : 100	1 : 10
CD44-PE	BD	Hu, Rt, Mu	Mono	—	Mouse	—	1 : 100	1 : 10
C-peptide	Cell signalling	Hu, Rt, Mu	Poly	4	Rabbit	—	1 : 100	NA
Insulin-Alexa 548	Santa Cruz	Hu, Rt, Mu	Mono	6	Guinea Pig	—	1 : 200	NA
Glucagon	Sigma	Hu, Rt, Mu	Mono	3.5	Mouse	—	1 : 200	NA
Somatostatin	Sigma	Hu, Mu	Poly	12	Rabbit	—	1 : 200	NA

**Table 2:** List of primer sequences of RT-PCR with annealing temperature and amplicon size.

Name of gene	Gene accession number	Primer sequence forward	Primer sequence reverse	PCR conditions (Tm)	Amplicon size (BP)
*α*-SMA	NT_039687.7	AGTCGCCATCAGGAACCTCGAG	ATCTTTTCCATGTCGTCCCAGTTG	60	296
Vimentin	NT_039202.7	AGCGGGACAACCTGGCCG	GGGAAGAAAAGGTTGGCAGAGGC	58	744
Nestin	NT_039240.7	GCGGGGCGGTGCGTGACTAC	AGGCAAGGGGGAAGAGAAGGATGT	58	326
PDX-1	NT_039324.7	CTC GCT GGG AAC GCT GGA ACA	GCT TTG GTG GAT TTC ATC CAC GG	55	229
Ngn-3	NT_039500.7	ACTAGGATGGCGCCTCATCCCTTG	GGTCTCTTCACAAGAAGTCTGAGA	57	658
Pax-4	NM_011038.2	TGGCTTCCTGTCCTTCTGTGAGG	TCCAAGACTCCTGTGCGGTAGTAG	62	214
Nkx-6.1	NM_144955.2	ATGGGAAGAGAAAACACACCAGAC	TAATCGTCGTCGTCCTCCTCGTTC	60	280
Insulin	NM_008386.3	GCCCAGGCTTTTGTCAAACA	CTCCCCACACACCAGGTAGAG	55	90
Glucagon	NM_008100.3	ATGAAGACCATTTACTTTGTGGCT	GGTGTTCATCAACCACTGCAC	58	243
Reg-1	NM_009042.1	AAGCTGAAGAAGACCTGCCA	TGTTAGGAGACCCAGTTGCC	60	123
GAPDH	NT_166349.1	CAAGGTCATCCATGACAACTTTG	GTCCACCACCCTGTTGCTGTAG	58	496

List of primer sequences for Q-PCR with annealing temperature and amplicon size					

PDX-1	NT_039324.7	ACTTGAGCGTTCCAATACGG	GCTTTGGTGGATTTCATCCACGG	55	813
INS-2	NM008387	TGTCTCTGGGGAAATGGGATTC	TGCTGCTTGACAAAAGCCTG	59	243

## Abbreviations

*E. littorale:*: *Enicostemma littorale*
ILCC: Islet-like cell clusters
SFM: Serum free media
ITS: Insulin transferrin selenite cocktail
DTZ: Dithizone
STZ: Streptozotocin
FBG: Fasting blood glucose
T1D: Type1 diabetes
T2D: Type2 diabetes
HGF: Hepatocyte growth factor
SMA: Smooth muscle actin
NaF: Sodium fluoride
ESI-MS/MS: Electron stimulated ionization mode for mass spectroscopy
INGAP: Insulin-like neurogenic growth peptide
NIDDM: Noninsulin-dependent diabetes mellitus.
