# Supplementary material for: A Small Molecule Swertisin from Enicostemma littorale Differentiates NIH3T3 Cells into Islet-Like Clusters and Restores Normoglycemia upon Transplantation in Diabetic Balb/c Mice
Source: Evid Based Complement Alternat Med. 2013 Apr 15;2013:280392. doi: 10.1155/2013/280392 (PMC3639639; doi:10.1155/2013/280392)
Supplement: Supplementary file 1 — Figure SP-1: represents densitometric quantification of vimentin protein expression and p-Smad-2 protein expression in 0-8 day time course in various NIH3T3 differentiation islet like clusters groups. Data is represented as Mean Band Density normalized to endogenous control beta actin. Figure SP-2: representing comparative flowcytometery quantification data of islet cell markers like insulin, c-peptide, glucagon from differentiated NIH3T3 islet like clusters and normal mouse islet cells. All three groups control SFM, Activin-A and Swertisin are compared to normal mouse islets cells. [file 280392.f1.pdf]

Legends to Supplementary figures:

Figure SP-1: represents densitometric quantification of vimentin protein expression and p-Smad-2 protein expression in 0-8 day time course in various NIH3T3 differentiation islet like clusters groups.

Figure SP-2: representing comparative flowcytometery quantification data of islet cell markers from differentiated NIH3T3 islet like clusters and normal mouse islet cells.

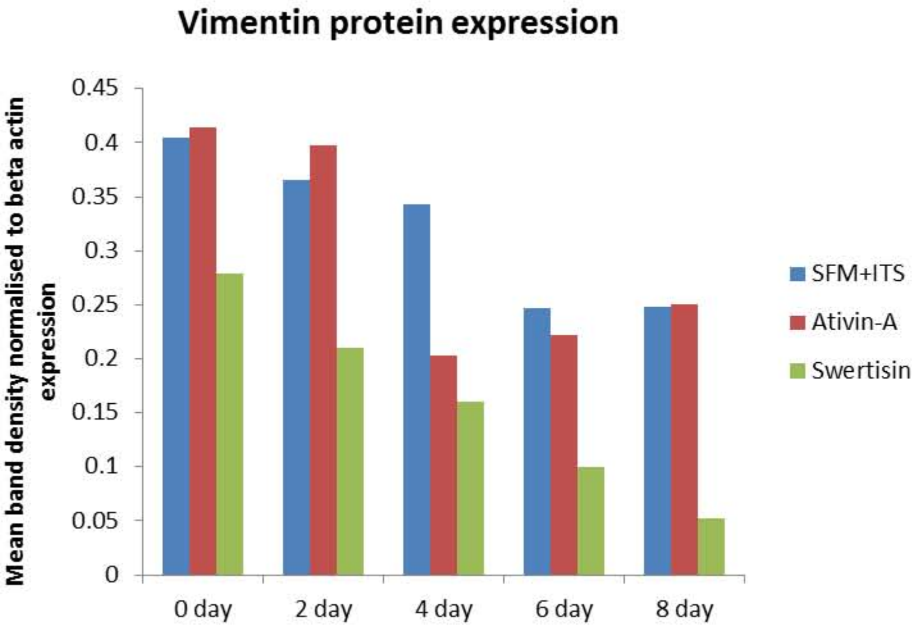
Vimentin protein expression
Mean band density normalised to beta actin expression
0.45
0.4
0.35
0.3
0.25
0.2
0.15
0.1
0.05
0
0 day
2 day
4 day
6 day
8 day
SFM+ITS
Ativin-A
Swertisin

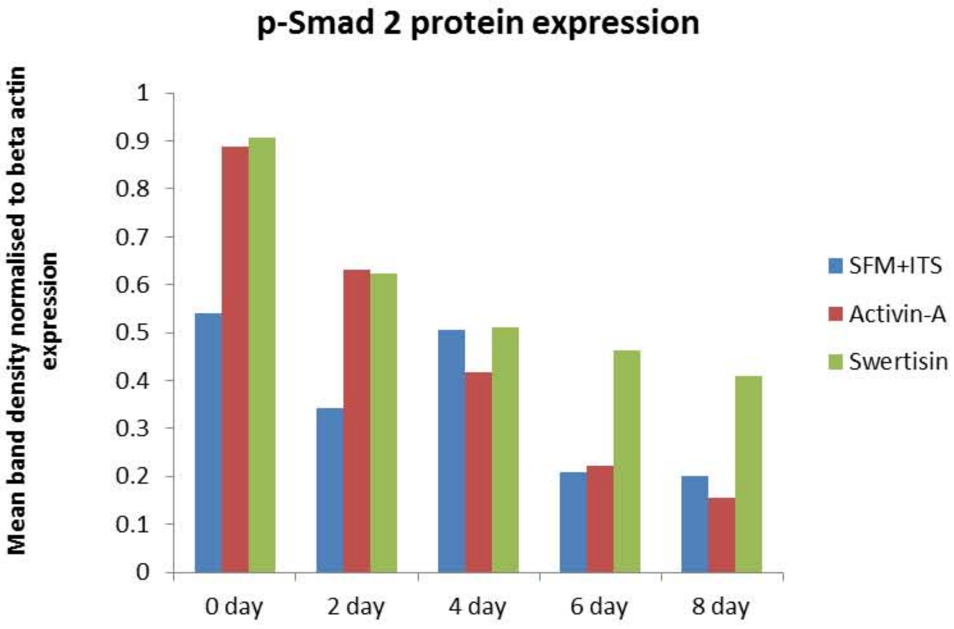
p-Smad 2 protein expression
Mean band density normalised to beta actin expression
1
0.9
0.8
0.7
0.6
0.5
0.4
0.3
0.2
0.1
0
0 day
2 day
4 day
6 day
8 day
SFM+ITS
Activin-A
Swertisin

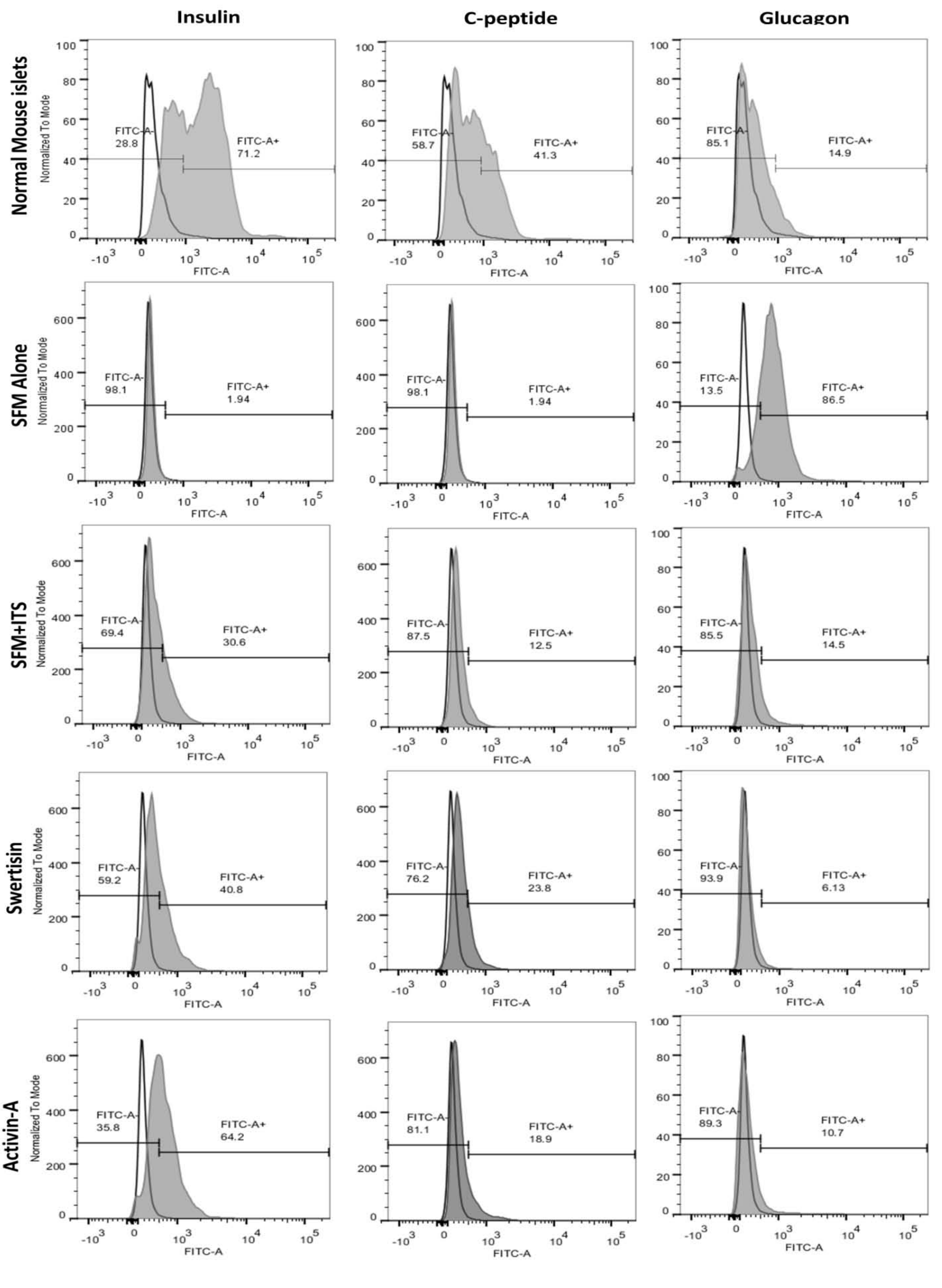
Insulin
C-peptide
Glucagon
Normal Mouse islets
SFM Alone
SFM+ITS
Swertisin
Activin-A
Normalized To Mode
FITC-A
FITC-A- 28.8
FITC-A+ 71.2
FITC-A- 58.7
FITC-A+ 41.3
FITC-A- 85.1
FITC-A+ 14.9
FITC-A- 98.1
FITC-A+ 1.94
FITC-A- 98.1
FITC-A+ 1.94
FITC-A- 13.5
FITC-A+ 86.5
FITC-A- 69.4
FITC-A+ 30.6
FITC-A- 87.5
FITC-A+ 12.5
FITC-A- 85.5
FITC-A+ 14.5
FITC-A- 59.2
FITC-A+ 40.8
FITC-A- 76.2
FITC-A+ 23.8
FITC-A- 93.9
FITC-A+ 6.13
FITC-A- 35.8
FITC-A+ 64.2
FITC-A- 81.1
FITC-A+ 18.9
FITC-A- 89.3
FITC-A+ 10.7
